# Is Pseudo-progression post-CAR T therapy real?

**DOI:** 10.46989/001c.160121

**Published:** 2026-05-12

**Authors:** Alicia Shin, Kian Rahbari, Wichai Chinratanalab, Bipin Savani, Adetola Kassim, James Jerkins, Bhagirathbhai Dholaria, Lindsay Orton, Rachel Gilmore, Kendall Shultes, Jana Sawyer, Salyka Sengsayadeth, Olalekan Oluwole, Tae Kon Kim

**Affiliations:** 1 University of California, Los Angeles, CA 90024, USA; 2 Division of Hematology/Oncology, Department of Medicine, Vanderbilt University Medical Center, Nashville, TN 37232, USA; 3 Vanderbilt-Ingram Cancer Center (VICC), Nashville, TN 37232, USA; 4 Veterans Affairs Medical Center, Tennessee Valley Healthcare System, Nashville, TN 37232, USA; 5 Department of Pathology, Microbiology, and Immunology, Vanderbilt University Medical Center, Nashville, TN 37232, USA

**Keywords:** CAR T cell therapy, pseudoprogression

## Case Presentation

### Case 1

**Figure 1.**
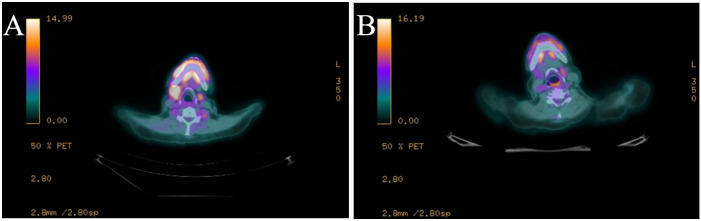
Right cervical pseudoprogression in diffuse large B-cell lymphoma after axicabtagene ciloleucel. PET/CT scan on day +30 showing a highly avid right cervical node at station IB measuring 2.2 x 1.5cm. (B) PET/CT from day +121 showing significant size and avidity reduction of the cervical node. Residual avidity consistent with prior disease

A 59-year-old woman with stage IV, double-hit diffuse large B-cell lymphoma was considered for chimeric antigen receptor T cell therapy (CAR T-cell therapy) after biopsy of a highly avid right axillary lymph node confirmed her disease had been refractory to five cycles of induction therapy with rituximab, dose-adjusted etoposide, prednisone, vincristine, cyclophosphamide, and doxorubicin (DA-R-EPOCH). She received bridging therapy with polatuzumab-vedotin and rituximab (Pola-R) for two cycles but developed clinical progression with an enlarging right cervical node; so, her bridging therapy was changed to rituximab, cyclophosphamide, vincristine, doxorubicin, and dexamethasone (R-hyperCVAD part A). She then proceeded with lymphodepleting chemotherapy, followed by infusion of axicabtagene ciloleucel. Her course was complicated by grade 1 cytokine release syndrome (CRS) requiring hospitalization from days +4 to +7 as well as grade 1 immune effector cell associated neurotoxicity (ICANs) requiring hospitalization from days +15 to +22. On day +30, she underwent a PET/CT (**Figure 1A**), which showed decreased size but increased FDG avidity in the cervical node (2.2 x 1.5 cm, previously 3.4 x 2.9 cm, SUV 8, previously 6.8). The scan was otherwise remarkable for unchanged minimally avid left supraclavicular and bilateral axillary nodes and moderately avid bilateral hilar nodes. A biopsy by fine needle aspirate of the right cervical node (highest FDG avidity) was consistent with necro-inflammatory cells including macrophages, neutrophils, and rare small lymphocytes, without any evidence of malignancy. Follow-up PET/CT on day +121 (**Figure 1B**) showed a further decrement in size of the right cervical node with no remaining avidity and no other findings of active lymphoma, consistent with a complete remission. She maintained her remission for the remainder of her life, with 750 days of disease-free survival.

### Case 2

**Figure 2.**
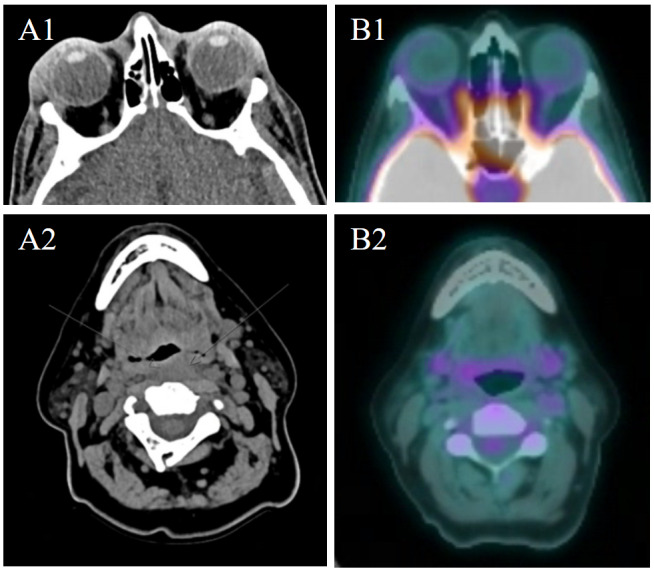
Lacrimal and retropharyngeal pseudoprogression in mantle cell lymphoma after brexucabtagene autoleucel. (A1) CT maxillofacial/neck on Day +11 showing symmetric lacrimal gland enlargement consistent with disease involvement. (A2) CT from Day +11 with arrows pointing to new retropharyngeal, poorly defined mass concerning for progression. (B1 and B2) PET CT on Day +30 demonstrating complete metabolic remission.

A 64-year-old woman with stage IVB mantle cell lymphoma previously achieving complete remission after induction with bendamustine and rituximab was considered for CAR T-cell therapy after she developed relapsed disease which was refractory to zanubrutinib. She was referred for CAR T-cell therapy evaluation and received R-HyperCVAD as bridging therapy, along with intrathecal methotrexate and cytarabine followed by apheresis. While off therapy awaiting CAR-T, she developed bilateral erythematous periorbital edema. The edema did not improve after ophthalmic and systemic antibiotics, so an MRI was obtained which showed enlargement of the lacrimal glands indicative of lymphomatous involvement. She proceeded with lymphodepleting chemotherapy followed by brexucabtagene autoleucel. Her course was complicated by grade 1 CRS requiring admission from days +9 to +15, managed with tocilizumab, and grade 2 ICANS requiring admission from days +16 to +18, which was treated with dexamethasone. During her admission for CRS, she also experienced worsening periorbital edema, so a maxillofacial and neck MRI was obtained on day +11. This showed unchanged enlarged lacrimal glands but newly revealed an ill-defined retropharyngeal thickening, suspicious for new disease, as well as a mildly enlarged left cervical level IV lymph node (**Figure 2)**. A fine needle aspiration of the left cervical node was performed, revealing scant lymphocytes and background fibrous tissue without definite evidence of lymphoma. Her periorbital edema resolved during that same admission, and she did not develop any palpable neck masses. On her day +30 PET/CT scan (**Figure 2B**), she demonstrated a complete response, with no avid lesions and decreased size of prior involved nodes and spleen. She maintained her complete response but developed prolonged hematologic toxicity requiring transfusions, intravenous immunoglobulin, and iron chelation. She passed away from pneumonia the following year, after 383 days of disease-free survival.

## PERSPECTIVE

### Pseudoprogression after CAR T-cell therapy

CAR T-cell therapy is a personalized immunotherapy wherein the patient’s T cells are engineered to selectively target tumor cells.^1–3^ The CAR is a synthetic cell receptor that binds to a target cell surface antigen, allowing cytotoxic immune cells to target antigen-expressing cells.^3,4^ CAR T-cell therapy has become a hallmark of therapy in hematologic malignancies – improving outcomes in relapsed/refractory multiple myeloma and B-cell lymphomas – and shows further promise in primary central nervous system (CNS) lymphoma, solid tumors, and even autoimmune disease.^4^ However, it is costly, with prolonged manufacturing times often requiring the use of bridging therapies to stabilize the malignancy.^5^

Compared with other cancer treatments, the response to immunotherapy, including CAR T-cell therapy, is not clearly predictable. When tumors grow after exposure to immunotherapy, their pattern of growth can be categorized as either true progression, hyperprogression, or pseudoprogression. True progression is described as a continuous growth in tumor size which indicates that the treatment is ineffective, and that a different therapy might be necessary. Hyperprogession is characterized as an acceleration in tumor growth after immunotherapy, in which a halt in immunotherapy is needed.^6,7^ Lastly, pseudoprogression is observed as an initial increase followed by a decrease in tumor size during the course of immunotherapy.^8,9^ While true progression clearly informs healthcare providers that a different treatment is needed, the occurrences of hyperprogression and pseudoprogression complicate this decision.

Pseudoprogression has been known as an atypical response pattern observed with immune checkpoint inhibitors (i.e. CTLA-4 blockade, PD-1/PD-L1 blockade) in which radiographic imaging initially demonstrates an increase in tumor burden or the appearance of new lesions, followed by subsequent tumor regression without a change in therapy.^9^ It was first systematically described in patients treated with CTLA-4 blockade, ipilimumab for advanced melanoma, leading to the development of immune-related response criteria to better capture these atypical patterns.^10^ The reported incidence varies by tumor type but is generally low (approximately 5–10% in melanoma and less frequent in most other solid tumors).^11,12^

Pseudoprogression appears to be relatively prevalent after CAR T-cell therapy. Schroeder and colleagues observed that 8 out of 78 patients receiving CAR T-cell therapy for non-Hodgkin lymphoma (NHL) or multiple myeloma (MM) experienced pseudoprogression over a 46-month treatment period,^13^ and Danylesko and colleagues reported an incidence of 3 out of 56 patients with pseudoprogression after CD19 CAR T-cell therapy for NHL, accounting for 12 percent of incident progression in the cohort.^14^ Pseudoprogression has been linked to increased patient anxiety and decreased quality of life,^15,16^ and it has further been shown to lead to premature treatment discontinuation due to clinical uncertainty – as the current RECIST clinical criteria cannot differentiate pseudoprogression from true progression.^17,18^ Clinicians need effective tools for both patient education and clinical decision-making to mitigate the harms of pseudoprogression.

### Mechanisms of pseudoprogression

The mechanism of pseudoprogression remains an active area of research; however, there are several plausible hypotheses. First, pseudoprogression may be caused by delayed effectiveness of therapy, with tumor cells continuing to grow until there is activation of an effective adaptive immune response, which requires time to establish.^19^ Second, the influx of lymphocytes, macrophages, and other immune cells into the tumor micro-environment can be mistaken for tumor growth, particularly as, when activated, they could cause inflammation characterized by proliferation of immune cells and edema due to capillary leakage.^6^ For instance, immune infiltration of activated T-cells has been confirmed in numerous cases of pseudoprogression after checkpoint inhibition of solid tumors.^8,20–22^ Lastly, cellular debris may accumulate from cancer cell apoptosis and appear as growth due to recruitment of phagocytes.

In CAR T-cell therapy, the patient’s own CD4 helper T cells, CD8 cytotoxic T cells, or CD19 CAR T cells manufactured from CD4 and CD8 cells are engineered to provide anti-tumor treatments.^1,2,23–25^ These studies observed an expansion of CD4 and/or CD8 cells after patients received CAR T cells in a similar pattern to those receiving immune checkpoint inhibitor therapy. Schroeder and colleagues, for example, observed that patients with pseudoprogression after CAR T-cell therapy had an earlier peak in CAR T cell expansion than expected.^13^ These findings suggest that pseudoprogression can be related heavily with the overactivation and expansion of CD4 and CD8 T cells. Since CD8 are directly cytotoxic, they infiltrate near the tumor cells and, as CD4 provides cytokine support, the infiltration accelerates the expansion for both CD4 and CD8 around the tumor. In addition, as cytokines also recruit other immune cells, such as monocytes, into the tumor sites, they enables immune influx and inflammation of the tumor. This model bears striking parallels with the recently described CAR T complication of local immune effector cell associated toxicity syndrome (LICATS) described in autoimmune disease by Hagen and coauthors.^26^ In LICATS, organs previously affected by a B cell–mediated autoimmune disease developed transient localized dysfunction after exposure to anti-CD19 CAR T-cells. LICATS occurred a median of ten days after infusion, during the B cell aplasia phase in all patients, and biopsy of affected organs demonstrated infiltrating T cells and myeloid cells without B cells. The authors hypothesized a localized inflammatory process designed to cleanse the tissues of the rapidly killed B cells. An absence of systemic symptoms like fever and the lack of elevated acute phase reactants such as IL-6 distinguish LICANS from CRS. Similarly, Danylesko and colleagues demonstrated that the level of IL-8 was low in cases of pseudoprogression after CAR T-cell therapy for B-cell lymphoma, with higher IL-8 levels predicting true progression.^14^ The temporary inflammation of the tumor is seen as progression when screened, and when inflammation stops, the tumor size “decreases”, indicating that the patient had pseudoprogression.

### Conclusions and future directions

CAR T cell therapy has been gaining popularity for its success, but its implementation remains limited due to its life-threatening toxicities of CRS and ICANS which require specialized multidisciplinary care to manage, its ambiguous success rates in solid tumor cells, and its extremely high cost.^27–35^ In addition, CAR T cell therapy also causes pseudoprogression, a phenomenon where tumor cell size initially seems to grow after immunotherapy but soon decreases, showcasing anti-tumor response.^8,9^ Pseudoprogression poses the risk of premature termination of appropriate, costly therapy, and has also been shown to decrease patient quality of life, so it is essential for clinicians to provide effective counseling to mitigate these harms. Overactivation and expansion of CD4 or CD8 T cells during CAR T therapy may be related to pseudoprogression. This speculation was derived from previous studies that identified how pseudoprogression after immune checkpoint inhibitor therapy revealed infiltration in CD4 and CD8 T cells. Thus, it would be important to comprehensively assess the immune landscape of biopsied tissue and peripheral blood using advanced scientific techniques to understand the mechanisms of pseudoprogression in patients who undergo CAR T cell therapy and the risk factors.^13^ In addition, biomarkers predicting pseudoprogression, such as IL-8, may enable us to delineate pseudoprogression versus true progression that may allow earlier intervention.

**Table 1.** Published reports of pseudoprogression after CAR T-cell therapy

**Reference**	**Disease**	**CAR T product**	**Key Points**
Wang et al., 2019^36^	DLBCL (n=3)	Anti-CD19 (not specified)	3 of 19 patients in a retrospective single center cohort of lymphoma patients demonstrated significant enlargement of known disease sites after CAR T-cell infusion on D5, D5, and D4, respectively, followed by rapid spontaneous regression.
Danylesko et al., 2021^14^	DLBCL (n=1) PMBCL (n=2)	Anti-CD19 (NCT02772198)	3 of 56 patients on trial developed symptomatic pseudoprogression between D2 and D7 which significantly improved or resolved spontaneously by D30 PET-CT.
Huang et al., 2021^37^	B-ALL (n=1)	Anti-CD19 (not specified)	Extramedullary disease to leg showed MRI growth on D16 and resolved spontaneously by D45 with metabolic remission on PET-CT.
Cohen et al., 2022^38^	DLBCL (n=1)	axicabtagene ciloleucel	Palpable cervical adenopathy on D9, confirmed FDG avid on D15 PET-CT, with biopsy confirming no lymphoma and spontaneous improvement by D42.
Schroeder et al., 2023^13^	Non-Hodgkin B-cell lymphoma (n=7) Multiple myeloma (n=1)	Anti-CD19 (not specified) Anti-BCMA (not specified)	Single center retrospective cohort of 78 patients who received CAR T-cell therapy, identifying a 10% incidence of pseudoprogression. Defined pseudoprogression as tumor growth within 10 days of infusion with spontaneous regression without alternative explanation.
Sortais et al., 2023^39^	DLBCL (n=5) PMBCL (n=1)	Anti-CD19 (not specified)	6 of 115 patients experienced pseudoprogression, all within one week of infusion. 4 were symptomatic and 2 were asymptomatic. All patients had concurrent CRS.
Tisserand et al., 2023^40^	Non-Hodgkin B-cell lymphoma (n=13)	Anti-CD19 (not specified)	Retrospective single center cohort study demonstrating early progression of undetermined significance in 18 of 105 patients. 13 of 18 cases were due to pseudoprogression.
Frank et al., 2024^41^	Large B-cell lymphoma, CD19 negative (n=5)	Anti-CD22 (not named)	4 of 38 patients receiving novel anti-CD22 product observed to have new hypermetabolic on D28 PET-CT which resolved spontaneously. 1 additional patient developed a new hypermetabolic lesion 14 months after infusion, shown to be a granuloma on biopsy, which resolved spontaneously.
Zhao et al., 2025^42^	Gastric cancer (n=1)	Anti-Claudin18.2 (not specified)	D28 CT scan showed growth of liver metastases meeting criteria for progression which gradually decreased in size with decreasing tumor markers. D100 biopsy of liver mass showed a large amount of immune infiltration with no tumor cells. The mass temporarily enlarged a second time after CAR T reinfusion on D127.
Miyachi et al., 2026^43^	DLBCL	axicabtagene ciloleucel	Painless periorbital mass noted on D12 which spontaneously resolved by D17. PET-CT on D30 confirmed completed metabolic remission.

### Authors’ Contribution

Writing – original draft: Alicia Shin (Equal), Kian Rahbari (Equal), Tae Kon Kim (Equal). Writing – review & editing: Wichai Chinratanalab (Equal), Bipin Savani (Equal), Adetola Kassim (Equal), James Jerkins (Equal), Bhagirathbhai Dholaria (Equal), Lindsay Orton (Equal), Rachel Gilmore (Equal), Kendall Shultes (Equal), Jana Sawyer (Equal), Salyka Sengsayadeth (Equal), Olalekan Oluwole (Equal). Conceptualization: Olalekan Oluwole (Equal), Tae Kon Kim (Equal). Visualization: Olalekan Oluwole (Lead).

### Competing of Interest – COPE

TKK is a medical advisor for Aimmunelink, and a member for advisory board for Arog Phamaceutical but not relevant to this project.

### Ethical Conduct Approval – Helsinki – IACUC

Not applicable

### Informed Consent Statement

All authors and institutions have confirmed this manuscript for publication.

### Data Availability Statement

All are available upon reasonable request.
